# Delay-induced periodic phenomenon in a diffusive regulated logistic model

**DOI:** 10.1186/s40064-016-2958-y

**Published:** 2016-08-09

**Authors:** Kejun Zhuang, Gao Jia

**Affiliations:** 1 Business School, University of Shanghai for Science and Technology, Shanghai, 200093 China; 2 School of Statistics and Applied Mathematics, Anhui University of Finance and Economics, Bengbu, 233030 China; 3 College of Science, University of Shanghai for Science and Technology, Shanghai, 200093 China

**Keywords:** Logistic model, Feedback control, Hopf bifurcation, Reaction–diffusion system, Delay, 35K57, 35B10, 35B32

## Abstract

The diffusive logistic growth model with time delay and feedback control is considered. First, the well-posedness and permanence of solutions are discussed by using some comparison techniques. Then, the sufficient conditions for stability of nonnegative constant steady states are established, and the occurrence of Hopf bifurcation at positive steady state is performed. Next, the bifurcation properties are derived by computing the normal form on center manifold. Our results not only supplement but also generalized some existing ones. Finally, some numerical simulations show the feasibility of our theoretical analyses.

## Background

The classic logistic model

1 $$\begin{aligned} \frac{\text{d}N(t)}{\text{d}t}=rN(t)\left[ 1-\frac{N(t)}{K} \right] , \quad r,k\in (0,+\infty ) \end{aligned}$$

was first proposed by Verhulst in 1838. It can be utilized to describe the single–species growth and has been the basis of varieties of models in population ecology and epidemiology. For system (1) and its generalized forms, the significant results involve the asymptotic properties (Berezansky et al. 2004; Röst 2011), permanence and stability (Fan and Wang 2010; Chen et al. 2006), periodicity (Sun and Chen 2007) and almost periodicity (Yang and Yuan 2008) of solutions, Hopf bifurcation (Sun et al. 2007; Song and Yuan 2007; Song and Peng 2006; Chen and Shi 2012), traveling wave front (Zhang and Sun 2014), free boundary problem (Gu and Lin 2014), and so on. In addition, the Hopf bifurcation analyses for some diffusive predator–prey systems were also done (see Yang 2015; Yang and Zhang 2016a, b).

In particular, Gopalsamy (1993) considered the controlled delay system in the following form

2 $$\begin{aligned} \left\{ \begin{array}{l} \frac{\text{d}N(t)}{\text{d}t}=rN(t)\left[ 1-\frac{a_1N(t)+a_2N(t-\tau )}{K} -cu(t)\right] , \\ \frac{\text{d}u(t)}{\text{d}t}=bN(t-\tau ) -au(t), \end{array} \right. \end{aligned}$$

where all the coefficients and time delay $\tau$ are positive constants, *N*(*t*) is the number of individuals at time *t*, and variable *u*(*t*) denotes an indirect control variable (see Aizerman and Gantmacher 1964; Lefschetz 1965). They have derived the sufficient conditions to guarantee that the positive equilibrium solution is globally asymptotical stable.

Strictly speaking, spatial diffusion can not be ignored in studying the natural biological system (Murray 2003; Ghergu and Radulescu 2012). In the real world, most populations are moving and the densities are dependent of time and space. Therefore, diffusion should be taken into account in studying the basic logistic equation. However, there have been very few results on the influence of time delay on the reaction–diffusion logistic model with feedback control.

Inspired by the previous discussions, we mainly consider the reaction–diffusion system as follows:

3 $$\begin{aligned} \left\{ \begin{array}{l} \frac{\partial N(x,t)}{\partial t}=d_1 \varDelta N(x,t)+rN(x,t)\left[ 1-\frac{a_1N(x,t)+a_2N(x,t-\tau )}{K}-cu(x,t) \right] , \\ \frac{\partial u(x,t)}{\partial t}=d_2 \varDelta u(x,t)+bN(x,t-\tau )-au(x,t), \end{array} \right. \end{aligned}$$

where $$(x,t)\in \varOmega \times [0,+\infty )$$, $$\varOmega =(0,l\pi )$$.

The model (3) is considered with the initial value conditions as follows

4 $$\begin{aligned} N(x,t) =\eta _1(x,t)\ge 0,\quad u(x,t)=\eta _2(x,t)\ge 0,\quad x\in [0,l\pi ]\times [-\tau , 0]. \end{aligned}$$

We also assume that the model (3) is closed and there is no emigration or immigration across the boundary. Hence, the boundary conditions are considered as

5 $$\begin{aligned} \frac{\partial N}{\partial \nu }=\frac{\partial u}{\partial \nu }=0,\quad (x,t)\in \partial \varOmega \times [0,+\infty ), \end{aligned}$$

where $\partial /\partial \nu$ represents the outward normal derivative on the boundary $\partial \varOmega$.

In this paper, we develop a reaction–diffusion logistic model with time delay and diffusion, which makes up perfectly for the deficiencies of the previous literatures. The main objective is to explore the dynamics of system (3) by regarding $\tau$ as the bifurcation parameter. The structure of this paper is arranged as follows. In section “Preliminaries”, we derive the well–posedness of solutions and the permanence of the system. In section “Occurrence of the Hopf bifurcation”, we establish the existence of Hopf bifurcation. In section “Bifurcation properties”, we get the formulae for determining the Hopf bifurcation properties. In section “Numerical simulations”, we illustrate our theoretical results by some numerical simulations. Finally, we give some discussions and conclusions.

## Preliminaries

As we know, spatial diffusion and time delay do not change the number and locations of constant equilibria because of no-flux boundary conditions. Then system (3) has two nonnegative equlibria $E_0=(0,0)$ and $E^{*}=(N^{*}, u^{*})$, where

$$\begin{aligned} N^{*}=\frac{aK}{a(a_1+a_2)+bcK},\quad u^{*}=\frac{b}{a}N^{*}=\frac{bK}{a(a_1+a_2)+bcK}. \end{aligned}$$

### Well–posedness of solutions

Here, for problem (3)–(5), we devote ourselves to the existence, uniqueness, nonnegativity and boundedness of solutions.

#### **Theorem 1**

*For any given initial data satisfying the conditions* (4) *and boundary conditions* (5), *system* (3) *has a unique global solution of system and the solution maintains nonnegative and uniformly bounded for all*$t\ge 0$.

#### *Proof*

Using the similar methods in Hattaf and Yousfi (2015), Hattaf and Yousfi (2015), we can get the local existence and uniqueness of solution (*N*(*x*, *t*), *u*(*x*, *t*)) with $x\in \bar{\varOmega }$ and $t\in [0,T)$, where *T* is the maximal existence time of solution.

It is easy to find that $\mathbf {0}=(0,0)$ and $\mathbf {M}=(M_1,M_2)$ are a pair of coupled lower–upper solutions to problem (3)–(5), where

$$\begin{aligned} M_1&= \max \left\{ \frac{K}{a_1}, \sup _{-\tau \le s\le 0} \Vert \varphi _1(\cdot ,s) \Vert _{C(\bar{\varOmega },{\mathbb {R}})} \right\} ,\\ M_2&= \max \left\{ \frac{bM_1}{a}, \sup _{-\tau \le s\le 0} \Vert \varphi _2(\cdot ,s) \Vert _{C(\bar{\varOmega },{\mathbb {R}})} \right\} . \end{aligned}$$

By means of the comparison theorem, we can obtain that $0\le N(x,t)\le M_1$ and $0\le u(x,t)\le M_2$ for $x\in \bar{\varOmega }$ and $t\in [0, T)$. It is obvious that the upper bound of solution is independent of the maximal existence interval [0, *T*). It follows from the standard theory for semilinear parabolic systems (Wu 1996; Henry 1993) that the solution globally exists. The proof is complete. $\square$

### Dissipativeness and permanence

In the following, we will show that system (3) is permanent, which means that any nonnegative solution of (3) is bounded as $t\rightarrow +\infty$ for all $x\in \varOmega$.

#### **Theorem 2**

(Dissipativeness) *The nonnegative solution* (*N*, *u*) *of system* (3) *satisfies*

$$\begin{aligned} \limsup _{t\rightarrow +\infty } N(x,t)\le \frac{K}{a_1}, \ \ \limsup _{t\rightarrow +\infty } u(x,t)\le \frac{bK}{aa_1}. \end{aligned}$$

#### *Proof*

Based on the first equation in system (3), we get

$$\begin{aligned} \frac{\partial N(x,t)}{\partial t}-d_1\varDelta N(x,t)\le rN(x,t)\left( 1-\frac{a_1}{K}N(x,t) \right) \quad \text{ for } \quad (x,t)\in \varOmega \times [0,+\infty ). \end{aligned}$$

Then from the standard comparison principle of parabolic equations, we can easily get

$$\begin{aligned} \limsup _{t\rightarrow +\infty } N(x,t)\le \frac{K}{a_1}. \end{aligned}$$

For an arbitrary $\varepsilon _1>0$, we could get a positive constant $T_1$ such that for any $t\ge T_1$,

$$\begin{aligned} N(x,t)\le \frac{K}{a_1}+\varepsilon _1. \end{aligned}$$

Thus, for any $T\in [T_1+\tau ,+\infty )$, we have

$$\begin{aligned} \frac{\partial u(x,t)}{\partial t}-d_2\varDelta u(x,t)\le b\left( \frac{K}{a_1}+\varepsilon _1 \right) -au(x,t). \end{aligned}$$

This implies

$$\begin{aligned} \limsup _{t\rightarrow +\infty } u(x,t)\le \frac{bK}{aa_1} \end{aligned}$$

by comparison principle of parabolic equations and the arbitrariness of $\varepsilon _1$. $\square$

#### **Theorem 3**

*If*$aa_1>aa_2+bcK$, *then system* (3) *is permanent.*

#### *Proof*

From Theorem 2, for an arbitrary $\varepsilon _2>0$, we can find a constant $T>T_1+T_2$, such that

$$\begin{aligned} u(x,t)\le \frac{bK}{aa_1}+\varepsilon _2 \end{aligned}$$

in $\varOmega \times [T_2,+\infty )$. Moreover, we can obtain

$$\begin{aligned}&\frac{\partial N(x,t)}{\partial t}-d_1\varDelta N(x,t) \\&\quad \ge rN(x,t)\left[ 1-\frac{a_2}{K}\left( \frac{K}{a_1}+\varepsilon _1 \right) -c\left( \frac{bK}{aa_1}+\varepsilon _2 \right) -\frac{a_1}{K}N(x,t) \right] , \end{aligned}$$

the comparison principle shows that

$$\begin{aligned} \liminf _{t\rightarrow +\infty } N(x,t)\ge \frac{K}{a_1}\frac{aa_1-aa_2-bcK}{aa_1}>0 \end{aligned}$$

due to the continuity as $\varepsilon _1 \rightarrow 0$ and $\varepsilon _2 \rightarrow 0$.

Similarly, we can also have

$$\begin{aligned} \liminf _{t\rightarrow +\infty } u(x,t)\ge \frac{bK}{aa_1}\frac{aa_1-aa_2-bcK}{aa_1}>0. \end{aligned}$$

Combining the results in Theorem 2, we can easily conclude that system (3) is permanent.

## Occurrence of the Hopf bifurcation

For system (3), we shall study the local stability of two constant steady states and the occurrence of Hopf bifurcation phenomenon through discussing the distribution of characteristic values.

Denote

$$\begin{aligned} u_1(t)=N(x,t),\quad u_2(t)=u(x,t),\quad U(t)=(u_1(t),u_2(t))^T. \end{aligned}$$

By defining the phase space ${\mathcal {C}}=C([-\tau ,0],X)$, we can rewritten system (3) as the semilinear functional differential equation:

6 $$\begin{aligned} \dot{U}(t)=D\varDelta U(t)+G(U_t), \end{aligned}$$

where $X=\{ (u,v)\in H^2(0,l\pi )\times H^2(0,l\pi )|u_x=v_x=0,x=0,l\pi \}$, $U_t(\cdot )=U(t+\cdot )$, $D=\text{ diag } \{d_1,d_2\}$, $\varDelta =\text{ diag }\{ \partial ^2/\partial x^2, \partial ^2/\partial x^2 \}$, and $G(U_t):{\mathcal {C}}\rightarrow X$ is defined by

$$\begin{aligned} G(U_t)=\left( \begin{array}{c} ru_1(t)\left( 1-\frac{a_1u_1(t)+a_2u_1(t-\tau )}{K}-cu_2(t) \right) \\ bu_1(t-\tau )-au_2(t) \end{array}\right) . \end{aligned}$$

The linear system of (6) at $E_0(0,0)$ is

7 $$\begin{aligned} \dot{U}(t)=D\varDelta U(t)+L_{E_0}(U_t), \end{aligned}$$

where

$$\begin{aligned} L_{E_0}(\varphi )=\left( \begin{array}{cc} r\varphi _1(0) &{} \quad 0 \\ b\varphi _1(-\tau ) &{} \quad -a\varphi _2(0) \end{array}\right) \end{aligned}$$

for $\varphi (\theta )=U_t(\theta )$, $\varphi =(\varphi _1,\varphi _2)^T\in {\mathcal {C}}$. The characteristic equation of (7) is

8 $$\begin{aligned} \lambda y-D\varDelta y-L_{E_0}(e^{\lambda \cdot y})=0, \end{aligned}$$

where $y\in \text{ dom }(\varDelta )\backslash \{0\}$, $\text{ dom }\varDelta \subset X$ and $e^{\lambda \cdot }(\theta )y=e^{\lambda \theta }y$ for $\theta \in [-\tau ,0]$. We know that the operator $\varDelta$ in $\varOmega$ with homogeneous Neumann boundary condition has the eigenvalues $-n^2/l^2$ and the corresponding eigenfunctions $\cos (nx/l)$, $n\in {\mathbb {N}}_0=\{0,1,2,\ldots \}$. By using the Fourier expansion in (8),

$$\begin{aligned} y=\sum _{n=0}^\infty \left( \begin{array}{c} \alpha _n \\ \gamma _n \end{array}\right) \cos (nx/l), \end{aligned}$$

where $\alpha _n$, $\gamma _n\in {\mathbb {C}}$. Therefore, the characteristic equation (8) can be transferred into

$$\begin{aligned} \left| \begin{array}{cc} \lambda +d_1\frac{n^2}{l^2}-r &{} \quad 0 \\ -be^{-\lambda \tau } &{} \quad \lambda +d_2\frac{n^2}{l^2}+a \end{array}\right| =0,\quad n\in {\mathbb {N}}_0 . \end{aligned}$$

We then obtain the characteristic values as follows

$$\begin{aligned} \lambda _{1,n}=-d_1\frac{n^2}{l^2}+r,\quad \lambda _{2,n}=-d_2\frac{n^2}{l^2}-a,\quad n\in {\mathbb {N}}_0. \end{aligned}$$

It is obvious that $\lambda _{1,0}=r>0$, and we can establish the instability of $E_0$.

### **Theorem 4**

*The trivial equilibrium*$E_0$*of system* (3) *is always unstable.*

Next, we will focus on the occurrence of Hopf bifurcation phenomenon.

Linearizing system (3) at $E^{*}=(N^{*}, u^{*})$, we get

9 $$\begin{aligned} \dot{U}(t)=D\varDelta U(t)+L(U_t), \end{aligned}$$

where $L:{\mathcal {C}}\rightarrow X$ is given by

$$\begin{aligned} L(\varphi )=\left( \begin{array}{cc} -\frac{ra_1}{K}N^{*}\varphi _1(0)-\frac{ra_2}{K}N^{*}\varphi _1(-\tau ) &{} \quad -cN^{*}\varphi _2(0) \\ b\varphi _1(-\tau ) &{} \quad -a\varphi _2(0) \end{array}\right) \end{aligned}$$

with $\varphi (\theta )=U_t(\theta )$, $\varphi =(\varphi _1,\varphi _2)^T\in {\mathcal {C}}$. Similar to the previous discussion, we can obtain the characteristic equation

10 $$\begin{aligned} \lambda ^2+A_n\lambda +B_n+e^{-\lambda \tau }(C\lambda +D_n)=0, \quad n\in {\mathbb {N}}_0, \end{aligned}$$

where

$$\begin{aligned} A_n&= (d_1+d_2)\frac{n^2}{l^2}+a+\frac{ra_1}{K}N^{*}>0,\\ B_n&= d_1d_2\frac{n^4}{l^4}+\left( a+\frac{ra_1}{K}N^{*} \right) d_1\frac{n^2}{l^2}+\frac{raa_1}{K}N^{*}>0,\\ C&= \frac{ra_2}{K}N^{*}>0,\\ D_n&= \frac{ra_2}{K}N^{*} d_1\frac{n^2}{l^2}+\frac{raa_2}{K}N^{*}+bcN^{*}>0. \end{aligned}$$

For $\tau =0$, Eq. (10) can be reduced to

$$\begin{aligned} \lambda ^2+(A_n+C)\lambda +B_n+D_n=0 \end{aligned}$$

with $A_n+C>0$ and $B_n+D>0$. On the basis of Routh–Hurwitz stability criterion, we can obtain the local stability of $E^{*}$ when $\tau =0$.

### **Lemma 1**

*The positive equilibrium is always locally asymptotically stable without time delay.*

### *Remark 1*

From Lemma 1, we can find that there is no Turing instability without time delay.

For $\tau \ne 0$, let us suppose that $\lambda =i\omega (\omega >0)$ satisfies Eq. (10).

First, plugging $\lambda =i\omega$ into Eq. (10) and then segregating the real and imaginary components with the help of Euler’s formula, we can get the following two equations of $\omega$

$$\begin{aligned} \left\{ \begin{array}{l} \omega ^2-B_n= D_n\cos \omega \tau +C\omega \sin \omega \tau , \\ -\omega A_n=C\omega \cos \omega \tau -D_n\sin \omega \tau . \end{array}\right. \end{aligned}$$

Second, solving these equations, we can obtain

11 $$\begin{aligned} \left\{ \begin{array}{l} \cos \omega \tau =\frac{(D_n-A_nC)\omega ^2-B_nD_n}{C^2\omega ^2+D_n^2} , \\ \sin \omega \tau =\frac{C\omega ^3+(A_nD_n-B_nC)\omega }{C^2\omega ^2+D_n^2}. \end{array}\right. \end{aligned}$$

Third, squaring both sides of those two equations and then adding them up, we get the following equation

12 $$\begin{aligned} \omega ^4+\left( A_n^2-2B_n-C^2\right) \omega ^2+B_n^2-D_n^2=0, \end{aligned}$$

where

$$\begin{aligned} A_n^2-2B_n-C^2&= \left( d_1^2+d_2^2\right) \frac{n^4}{l^4}+2\left( \frac{ra_1}{K}N^{*} d_1+ad_2 \right) \frac{n^2}{l^2}+\frac{r^2\left( a_1^2-a_2^2\right) }{K^2}N^{*2} ,\\ B_n^2-D_n^2&= (B_n+D_n)\left( d_1d_2\frac{n^4}{l^4}+\left( a+\frac{ra_1}{K}N^{*}\right) d_1\frac{n^2}{l^2} +\frac{raa_1}{K}N^{*} -\frac{ra_2}{K}N^{*} d_1\frac{n^2}{l^2}\right. \\&\quad\left. -\frac{raa_2}{K}N^{*}-bcN^{*} \right) . \end{aligned}$$

### **Lemma 2**

*For*$\tau >0$, *we have*

*(i) If*$a_1>a_2+\frac{bcK}{ar}$, *then Eq.* (10) *does not have purely imaginary root.*

*(ii) If*$a_2<a_1<a_2+\frac{bcK}{ar}$, *then there exists*$N_0 \in {\mathbb {N}}_0$, *such that Eq.* (10) *does not have purely imaginary root when*$n>N_0$, *and has a pair of conjugate purely imaginary eigenvalues when*$0\le n\le N_0$.

### *Proof*

We can easily verify that $A_n^2-2B_n-C^2>0$ and $B_n^2-D_n^2>0$ when $a_1>a_2+\frac{bcK}{ar}$. This means that Eq. (12) has no positive root. In other words, there could be no purely imaginary root in Eq. (10) for any $\tau >0$.

On the contrary, if $a_2<a_1<a_2+\frac{bcK}{ar}$, then $B_0^2-D_0^2<0$ and there exists $N\in {\mathbb {N}}_0$ such that

$$\begin{aligned} \left\{ \begin{array}{ll} B_n^2-D_n^2<0, &{} \quad n=0,1,2,\ldots , N_0, \\ B_n^2-D_n^2\ge 0, &{} \quad n=N_0+1,N_0+2, \ldots . \end{array}\right. \end{aligned}$$

That is to say, Eq. (12) has no positive root when $n>N_0$ and has the unique positive root $\omega _n$ when $0\le n \le N_0$, where

$$\begin{aligned} \omega _n=\left( \frac{-(A_n^2-2B_n-C^2)+\sqrt{(A_n^2-2B_n-C^2)^2-4(B_n^2-D_n^2)}}{2} \right) ^{\frac{1}{2}}. \end{aligned}$$

By direct computation, we have

$$\begin{aligned} A_nD_n-B_nC&= \frac{ra_2}{K}N^{*} d_2^2\frac{n^4}{l^4}+\left( bcN^{*} d_1+\frac{2ra_2}{K}N^{*} d_2 +bcN^{*} d_2 \right) \frac{n^2}{l^2}\\&\quad+\frac{ra^2a_2}{K}N^{*}+abcN^{*}+\frac{ra_1bc}{K}N^{*2} \\&> 0. \end{aligned}$$

Moreover, Eq. (10) has characteristic values $\pm i\omega _n$ with

$$\begin{aligned} \tau _j^{(n)}=\tau _0^{(n)}+\frac{2j\pi }{\omega _n}, \quad 0\le n\le N_0, \quad j=0,1,2,\ldots , \end{aligned}$$

where

$$\begin{aligned} \tau _0^{(n)}=\frac{1}{\omega _n}\arccos \frac{(D_n-A_nC)\omega _n^2-B_nD_n}{D_n^2+C^2\omega _n^2}. \end{aligned}$$

This completes the proof. $\square$

We now check the transversality condition.

### **Lemma 3**

*If*$a_2<a_1<a_2+\frac{bcK}{ar}$, *then*$\left. \frac{\text{ dRe }(\lambda ) }{\text{d}\tau }\right| _{\tau =\tau _j^{(n)}}>0$*for*$j\in {\mathbb {N}}_0$*and*$n\in \{ 0, 1, 2, \ldots , N_0 \}$ .

### *Proof*

By taking the derivatives on both sides of (10) with respect to $\tau$, we can get

$$\begin{aligned} 2\lambda \frac{\text{d}\lambda }{\text{d}\tau }+A_n\frac{\text{d}\lambda }{\text{d}\tau }+C e^{-\lambda \tau }\frac{\text{d}\lambda }{\text{d}\tau }+(C\lambda +D_n)e^{-\lambda \tau }\left( -\lambda -\tau \frac{\text{d}\lambda }{\text{d}\tau } \right) =0, \end{aligned}$$

and

$$\begin{aligned} \left( \frac{\text{ d}\lambda }{\text{ d}\tau } \right) ^{-1}&= \frac{2\lambda +A_n+Ce^{-\lambda \tau }-\tau e^{-\lambda \tau }(C\lambda +D_n)}{\lambda e^{-\lambda \tau }(C\lambda +D_n)} \\&= \frac{(2\lambda +A_n)e^{\lambda \tau }+C}{\lambda (C\lambda +D_n)}-\frac{\tau }{\lambda }. \end{aligned}$$

On the basis of (11) and (12), we get

$$\begin{aligned} \left( \frac{\text{ d}\lambda }{\text{d}\tau } \right) _{\tau =\tau _j^{(n)}}^{-1}&= \frac{(2i\omega _n+A_n)\left( \cos \omega _n\tau _j^{(n)}+i\sin \omega _n\tau _j^{(n)}\right) +C}{i\omega _n(iC\omega _n+D_n)} -\frac{\tau _j^{(n)}}{i\omega _n} \\&= \frac{C+A_n\cos \omega _n\tau _j^{(n)}-2\omega _n\sin \omega _n\tau _j^{(n)}}{-C\omega _n^2+iD_n\omega _n} -\frac{\tau _j^{(n)}}{i\omega _n} \\&\quad+ \frac{i\left( 2\omega _n\cos \omega _n\tau _j^{(n)}+A_n\sin \omega _n\tau _j^{(n)}\right) }{-C\omega _n^2+iD_n\omega _n} . \end{aligned}$$

Further simplification will lead to

$$\begin{aligned} \text{ Re }\left( \frac{\text{d}\lambda }{\text{d}\tau } \right) _{\tau =\tau _j^{(n)}}^{-1}&= \frac{D_n\omega _n\left( 2\omega _n\cos \omega _n\tau _j^{(n)}+A_n\sin \omega _n\tau _j^{(n)}\right) }{\left( C\omega _n^2\right) ^2+(D_n\omega _n)^2}\\&\quad-\frac{C\omega _n^2\left( C+A_n\cos \omega _n\tau _j^{(n)}-2\omega _n\sin \omega _n\tau _j^{(n)}\right) }{\left( C\omega _n^2\right) ^2+(D_n\omega _n)^2} \\&= \frac{\omega ^4_n+D_n^2-B_n^2}{\left( C\omega _n^2\right) ^2+\left( D_n\omega _n\right) ^2} \\&> 0. \end{aligned}$$

The proof is complete. $\square$

According to Lemmas 1–3 and the Hopf bifurcation theory developed by Wu (1996), the following conclusions can be drawn.

### **Theorem 5**

*Define*

$$\begin{aligned} \tau _0=\min _{n\in \{ 0,1,2,\ldots ,N_0 \}, j\in {\mathbb {N}}_0}\left\{ \tau _j^{(n)} \right\} . \end{aligned}$$

*(i) If*$a_1>a_2+\frac{bcK}{ar}$, *then for any*$\tau >0$, *the positive equilibrium*$E^{*}$*is always locally asymptotically stable.*

*(ii) If*$a_2< a_1< a_2+\frac{bcK}{ar}$, *then*$E^{*}$*is locally asymptotically stable when*$\tau \in [0,\tau _0)$, *and is unstable when*$\tau \in (\tau _0,+\infty )$.

*(iii) System* (3) *has a Hopf bifurcation from*$E^{*}$*at*$\tau _j^{(n)}$*with*$n\in \{ 0,1,2,\ldots ,N_0 \}$*and*$j\in {\mathbb {N}}_0$. *If*$n=0$, *the periodic solutions bifurcating positive equilibrium are all spatially homogeneous. Otherwise, these bifurcating periodic solutions are spatially inhomogeneous.*

## Bifurcation properties

In Theorem 5, we have demonstrated that there exist some spatially homogeneous or inhomogeneous periodic solutions when time delay crosses through some particular values. We are now in the position to investigate the bifurcation properties.

In general, we use $\tau ^{*}$ to denote an arbitrary value of $\tau _j^{(n)}$ with $j\in {\mathbb {N}}_0$ and $n\in \{0,1,2,\ldots ,N_0\}$. And we also use $\pm i\omega ^{*}$ to denote the corresponding simply purely imaginary roots $\pm i\omega _n$.

Set $\tilde{N}(\cdot ,t)=N(\cdot ,\tau t)$, $\tilde{u}(\cdot ,t)=u(\cdot ,\tau t)$, $\tilde{U}(t)=(\tilde{N}(\cdot ,t),\tilde{u}(\cdot ,t))$, and $\tau =\tau ^{*}+\alpha$ with $\alpha \in {\mathbb {R}}$. For simplicity we drop the tilde and rewrite system (3) as follows,

13 $$\begin{aligned} \frac{\text{d}U(t)}{\text{d}t}=\tau D\varDelta U(t)+L(\alpha )(U_t)+f(U_t,\alpha ), \end{aligned}$$

where $\varphi =(\varphi _1,\varphi _2)^T\in {\mathcal {C}}$, $L(\alpha )(\cdot ): {\mathcal {C}}\rightarrow X$ and $f: {\mathcal {C}}\times {\mathbb {R}}\rightarrow X$ are respectively denoted by

$$\begin{aligned} L(\alpha )(\varphi )=(\tau ^{*}+\alpha )\left( \begin{array}{c} -\frac{ra_1}{K}N^{*}\varphi _1(0)+\frac{ra_2}{K}N^{*}\varphi _1(-1)-cN^{*}\varphi _2(0)\\ b\varphi _1(-1)-a\varphi _2(0) \end{array}\right) \end{aligned}$$

and

$$\begin{aligned} f(\varphi ,\alpha )=(\tau ^{*}+\alpha )\left( \begin{array}{c} -\frac{2ra_1}{K}\varphi _1^2(0)-\frac{ra_2}{K}\varphi _1(0)\varphi _1(-1)-rc\varphi _1(0)\varphi _2(0) \\ 0 \end{array}\right) . \end{aligned}$$

Note that $\alpha =\tau -\tau ^{*}$, we can find that system (13) may causes a Hopf bifurcation when $\alpha =0$.

For the following linear differential equation:

14 $$\begin{aligned} \dot{U}(t)=\tau D\varDelta U(t)+L(\alpha )(U_t), \end{aligned}$$

we can easily deduce that the corresponding characteristic equation has characteristic values $\pm \text{ i }\omega ^{*}\tau ^{*}$ when $\alpha =0$.

Next, we discuss the following differential equation:

15 $$\begin{aligned} \dot{Y}(t)=-\tau D n^2 Y(t)+L(\alpha )(Y_t). \end{aligned}$$

We can use Riesz representation theorem here, which tells us that there is a $$2\times 2$$ matrix function $$\eta (\theta , \alpha )$$$$(-1\le \theta \le 0)$$ with bounded variation elements satisfying

$$\begin{aligned} -\tau D \frac{n^2}{l^2} \varphi (0)+L(\alpha )(\varphi )=\int _{-1}^0\text{d} [\eta (\theta , \alpha )]\varphi (\theta ), \end{aligned}$$

where

$$\begin{aligned} \eta (\theta ,\alpha )=\left\{ \begin{array}{ll} (\tau ^{*}+\alpha ) \left( \begin{array}{cc} -d_1\frac{n^2}{l^2}-\frac{ra_1}{K}N^{*} &{} -cN^{*} \\ 0 &{} -d_2\frac{n^2}{l^2}-a \end{array} \right) , &{} \quad \theta =0, \\ 0, &{} \quad \theta \in (-1,0),\\ (\tau ^{*}+\alpha )\left( \begin{array}{cc} -\frac{ra_2}{K}N^{*} &{} 0 \\ b &{} 0 \end{array} \right) ,&\quad \theta =-1. \end{array} \right. \end{aligned}$$

For $\varPhi \in C^1([-1,0],{\mathbb {R}}^2)$, $\varPsi \in C^1([0,1],{\mathbb {R}}^2)$, we define

$$\begin{aligned} A_1(\varPhi (\theta ))&= \left\{ \begin{array}{ll} \frac{\text{d}\varPhi (\theta )}{\text{d}\theta }, &{} \quad \theta \in [-1,0),\\ \int _{-1}^0[\text{d}\eta _0(\theta )]\varPhi (\theta ), &{} \quad \theta =0, \end{array} \right. \\ A_1^{*}(\varPsi (s))&= \left\{ \begin{array}{ll} -\frac{\text{d}\varPsi (s)}{\text{d}s}, &{} \quad s\in (0,1],\\ \int _{-1}^0[\text{d}\eta _0(\theta )]\varPsi (-\theta ), &{} \quad s=0. \end{array} \right. \end{aligned}$$

Then the formal adjoint, $A_1^{*}$, of $A_1$ is given by

$$\begin{aligned} (\varPsi ,\varPhi )_0&= \overline{\varPsi }(0)\varPhi (0)-\int _{-1}^0\int _{\zeta =0}^\theta \overline{\varPsi }(\zeta -\theta )\text{d}[\eta (\theta ,0)]\varPhi (\zeta )\text{d}\zeta \\&= \overline{\varPsi }(0)\varPhi (0)+\tau ^{*}\int _{-1}^0 \overline{\varPsi }(\zeta +1) \left( \begin{array}{cc} -\frac{ra_2}{K}N^{*} &{} 0 \\ b &{} 0 \end{array} \right) \varPhi (\zeta )\text{d}\zeta . \end{aligned}$$

By calculation, we can find that $q(\theta )=(1,\xi )^Te^{i\omega ^{*}\theta \tau ^{*}}$ and $q^{*}(s)=M(1,\eta )e^{i\omega ^{*} s \tau ^{*}}$ are eigenvectors of $A_1$ and $A_1^{*}$ associated with $i\omega ^{*}\tau ^{*}$, respectively, where

$$\begin{aligned} \theta \in [-1,0], \quad s\in [0,1], \end{aligned}$$

and

$$\begin{aligned} \xi =\frac{be^{-i\omega ^{*}\tau ^{*}}}{i\omega ^{*}+a}, \ \ \eta =\frac{cN^{*}}{i\omega ^{*}+a}, \ \ M=\left[ 1+\overline{\xi }\eta +\tau ^{*}\left( b\eta -\frac{ra_2}{K}N^{*} \right) e^{i\omega ^{*}\tau ^{*}} \right] ^{-1}. \end{aligned}$$

Then $P=span\{ q(\theta ),\overline{q(\theta )} \}$, $P^{*}=span\{ q^{*}(s),\overline{q^{*}(s)} \}$ are the center subspace of system (3).

Define $h\cdot f_n=h_1\beta _n^1+h_2\beta _n^2$, $f_n=\left( \beta _n^1,\beta _n^2\right)$ and $\beta _n^1=\left( \cos \frac{nx}{l},0\right) ^T$, $\beta _n^2=\left( 0,\cos \frac{nx}{l}\right) ^T$. The complex-valued $L^2$ inner product on Hilbert space $X_C$ are

16 $$\begin{aligned} \langle U_1,U_2 \rangle =\frac{1}{l\pi }\int _0^{l\pi } (u_1\overline{v_1}+u_2\overline{v_2})\text{d}x, \end{aligned}$$

for $$U_1=(u_1,u_2), U_2=(v_1,v_2)\in X_C$$. And $$\langle \beta _0^i, \beta _0^i \rangle =1$$, $$\langle \beta _n^i, \beta _n^i \rangle =\frac{1}{2}$$, $$i=1,2$$, $$n=1,2,\ldots$$,

17 $$\begin{aligned} \langle \varPhi , f_n \rangle =\left( \langle \varPhi , \beta _n^1 \rangle ,\langle \varPhi , \beta _n^2 \rangle \right) , \end{aligned}$$

where $\varPhi \in C([-1,0],X)$. We can establish the center subspace of system (14) at $\alpha =0$ as follows

18 $$\begin{aligned} P_{CN}\mathcal {L}=\left\{ (q(\theta )z+ \overline{q(\theta )}\bar{z})\cdot f_n, z\in {\mathbb {C}}\right\} . \end{aligned}$$

Based on the conclusions drawn by Wu (1996) and Hassard et al. (1981), the solutions of (13) are

$$\begin{aligned} U_t=(q(\theta )z(t)+\overline{q(\theta )}\bar{z}(t))\cdot f_n+W(z(t),\bar{z}(t),\theta ), \end{aligned}$$

where

19 $$\begin{aligned} W(z,\bar{z},\theta )=W_{20}\frac{z^2}{2}+W_{11}z\bar{z}+W_{02}\frac{\bar{z}^2}{2}+\cdots . \end{aligned}$$

Moreover, for $U_t\in C_0$ of (13) at $\tau =\tau ^{*}$, we have $\dot{z}=i\omega ^{*}\tau ^{*} z+g(z,\bar{z})$, where

20 $$\begin{aligned} g(z,\bar{z})=\overline{q^{*}(0)} \langle f(U_t, 0), f_n \rangle =g_{20}\frac{z^2}{2}+g_{11}z\bar{z}+g_{02}\frac{\bar{z}^2}{2}+g_{21}\frac{z^2\bar{z}}{2}+\cdots . \end{aligned}$$

By (16)–(20), we can compute

$$\begin{aligned} g_{20}&= \left\{ \begin{array}{ll} 0, &{} \quad n=1,2,\ldots , \\ -2\tau ^{*}\overline{M}\left\{ \frac{ra_1}{K}+\frac{ra_2}{K}e^{-i\omega ^{*}\tau ^{*}}+rc\xi \right\} , &{} \quad n=0, \end{array} \right. \\ g_{11}&= \left\{ \begin{array}{ll} 0, &{} \quad n=1,2,\ldots ,\\ -2\tau ^{*}\overline{M}\left\{ \frac{ra_1}{K}+rc\text{ Re }\{\xi \}+\frac{ra_2}{K}\text{ Re }\{ e^{i\omega ^{*}\tau ^{*}} \} \right\} , &{} \quad n=0, \end{array}\right. \\ g_{02}&= \overline{g_{20}} ,\\ g_{21}&= -2\frac{\overline{M}\tau ^{*}}{l\pi }\left\{ \int _0^{l\pi } \frac{2ra_1}{K}(W_{11}^{(1)}(0)+W_{20}^{(1)}(0))\cos ^2\frac{nx}{l}\text{d}x \right. \\&\quad+\int _0^{l\pi } rc \left( W_{11}^{(2)}(0)+\frac{1}{2}W_{20}^{(2)}(0)+\frac{1}{2}\bar{\xi }W_{20}^{(1)}(0)+\xi W_{11}^{(1)}(0) \right) \cos ^2\frac{nx}{l}\text{d}x\\&\quad+\int _0^{l\pi } \frac{ra_2}{K}\left( e^{-i\omega ^{*}\tau ^{*}}W_{11}^{(2)}(0)+\frac{1}{2}e^{i\omega ^{*}\tau ^{*}}W_{20}^{(2)}(0)+\frac{1}{2}\bar{\xi }W_{20}^{(1)}{(-1)} \right) \cos ^2\frac{nx}{l}\text{d}x \\&\quad \left. + \int _0^{l\pi } \frac{ra_2}{K}\xi W_{11}^{(11)}(-1)\cos ^2\frac{nx}{l}\text{d}x \right\} . \end{aligned}$$

Then we should compute $W_{20}(\theta )$ and $W_{11}(\theta )$ to determine $g_{21}$. Following the formulas in Wu (1996), We can obtain that

$$\begin{aligned} W_{20}(\theta )&= \left( \frac{ig_{20}}{\omega ^{*}\tau ^{*}}q(\theta ) +\frac{i\overline{g_{02}}}{3\omega ^{*}\tau ^{*}}\overline{q(\theta )} \right) \cdot f_n+E_1 e^{2i\omega ^{*}\tau ^{*}\theta },\\ W_{11}(\theta )&= \left( -\frac{ig_{11}}{\omega ^{*}\tau ^{*}}1(\theta ) +\frac{i\overline{g_{11}}\overline{q(\theta )}}{\omega ^{*}\tau ^{*}} \right) \cdot f_n+E_2,\\ E_1&= E_1'\times \left( \begin{array}{c} -\frac{2ra_1}{K}-\frac{2ra_2}{K}e^{-i\omega ^{*}\tau ^{*}} -2rc\xi \\ 0 \end{array} \right) \cos ^2\frac{nx}{l},\\ E_1'&= \left( \begin{array}{cc} 2i\omega ^{*}+d_1\frac{n^2}{l^2}+\frac{ra_1}{K}N^{*}+\frac{ra_2}{K}N^{*} e^{-2i\omega ^{*}\tau ^{*}} &{} c N^{*} \\ -b e^{-2i\omega ^{*}\tau ^{*}} &{} 2i\omega ^{*}+a+d_2\frac{n^2}{l^2} \end{array} \right) ^{-1}, \end{aligned}$$

and

$$\begin{aligned} E_2&= E_2'\times \left( \begin{array}{c} -\frac{2ra_1}{K} -2rc\text{ Re }\{\xi \} -\frac{2ra_2}{K}\text{ Re }\{ e^{i\omega ^{*}\tau ^{*}} \} \\ 0 \end{array} \right) \cos ^2\frac{nx}{l},\\ E_2'&= \left( \begin{array}{cc} d_1\frac{n^2}{l^2}+\frac{ra_1}{K}N^{*}+\frac{ra_2}{K}N^{*} &{} c N^{*} \\ -b &{} a+d_2\frac{n^2}{l^2} \end{array} \right) ^{-1}. \end{aligned}$$

From the previous expressions of $g_{20}$, $g_{11}$, $g_{02}$ and $g_{21}$, we can further compute

$$\begin{aligned} c_1(0)&= \frac{i}{2\omega ^{*}\tau ^{*}}\left( g_{20}g_{11}-2|g_{11}|^2-\frac{1}{3}|g_{02}|^2 \right) +\frac{g_{21}}{2},\\ \mu _2&= -\frac{\text{ Re }(c_1(0))}{\text{ Re }(\lambda ' (\tau ^{*}))},\\ \beta _2&= 2\text{ Re }(c_1(0)),\\ T_2&= -\frac{1}{\omega ^{*}\tau ^{*}}(\text{ Im }(c_1(0))+\mu _2 \text{ Im }(\lambda ' (\tau ^{*}))). \end{aligned}$$

On account of preceding calculations, we arrive at the following conclusion on the bifurcation properties.

### **Theorem 6**

*The bifurcation direction is supercritical if*$\mu _2>0$, *which means that the periodic solution exists for*$\tau >\tau _0$. *On the contrary, the bifurcation direction is subcritical if*$\mu _2<0$, *which means that the periodic solution exists for*$\tau <\tau _0$.

*Moreover, the periodic solution is orbitally asymptotically stable if*$\beta _2<0$, *or unstable if*$\beta _2>0$. *The period of periodic solution is monotonically increasing at the time delay*$\tau$*when*$T_2>0$, *or is monotonically decreasing at the time delay*$\tau$*when*$T_2<0$.

## Numerical simulations

In this section, we give some numerical examples to test the preceding results with assistance of MATLAB.

For system (3), let $\varOmega =(0,2\pi )$ and choose

$$\begin{aligned} d_1=1, \quad d_2=0.5, \quad r=0.6, \quad a=b=c=1, \quad a_1=a_2=2, \quad K=1, \end{aligned}$$

and the initial values $N(x,0)=0.5$ and $u(x,0)=0.9$. Then we can get the positive equilibrium $E^{*}=(0.2,0.2)$. By direct computation, we have $N_0=0$, $\omega _0\approx 0.348266$, and $\tau _0^{(0)}\approx 5.81966$, then the Hopf bifurcation values are given by

$$\begin{aligned} \tau _j^{(n)}=\tau _j^{(0)}=\tau _0^{(0)}+\frac{2j\pi }{\omega _0}, \quad j=0,1,2,\ldots \end{aligned}$$

**Fig. 1:**
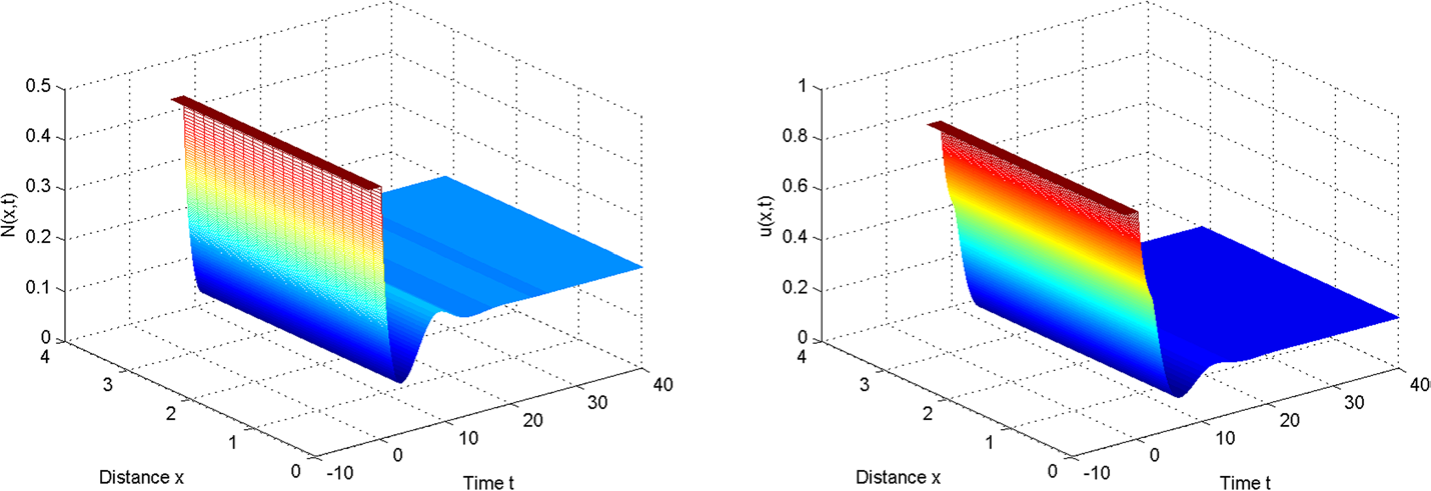
The equilibrium $E^{*}$ is stable when $\tau =2<\tau _0$

Concretely, $\tau _0=\tau _0^{(0)}\approx 5.81966$, $\tau _1^{(0)}\approx 23.861$, $\tau _2^{(0)}\approx 41.9024$, ... From Fig. 1, we can see the asymptotical stability of positive equilibrium $E^{*}$ when time delay is slightly smaller than the first bifurcation value $\tau _0$.

**Fig. 2:**
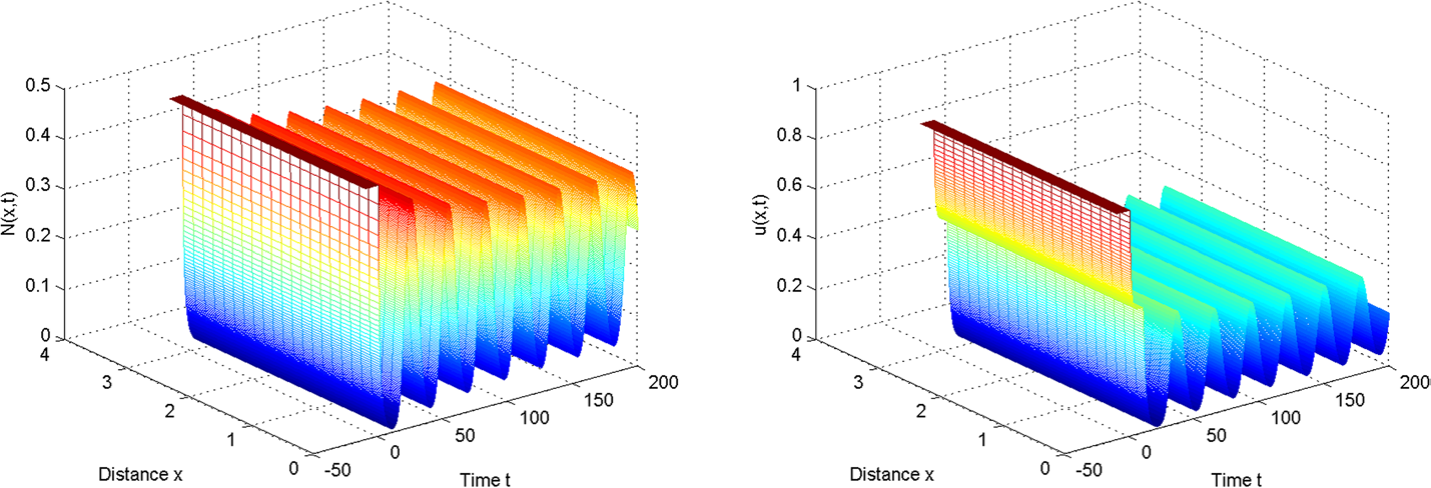
Spatially periodic solution exists when $\tau =10$

Moreover, we can obtain $c_1(0)\approx -1.4328+1.53343i$. From Theorem 6, the Hopf bifurcation is supercritical, that is, the periodic solutions exist for $\tau >\tau _0$, and they are orbitally asymptotically stable (see Fig. 2).

**Fig. 3:**
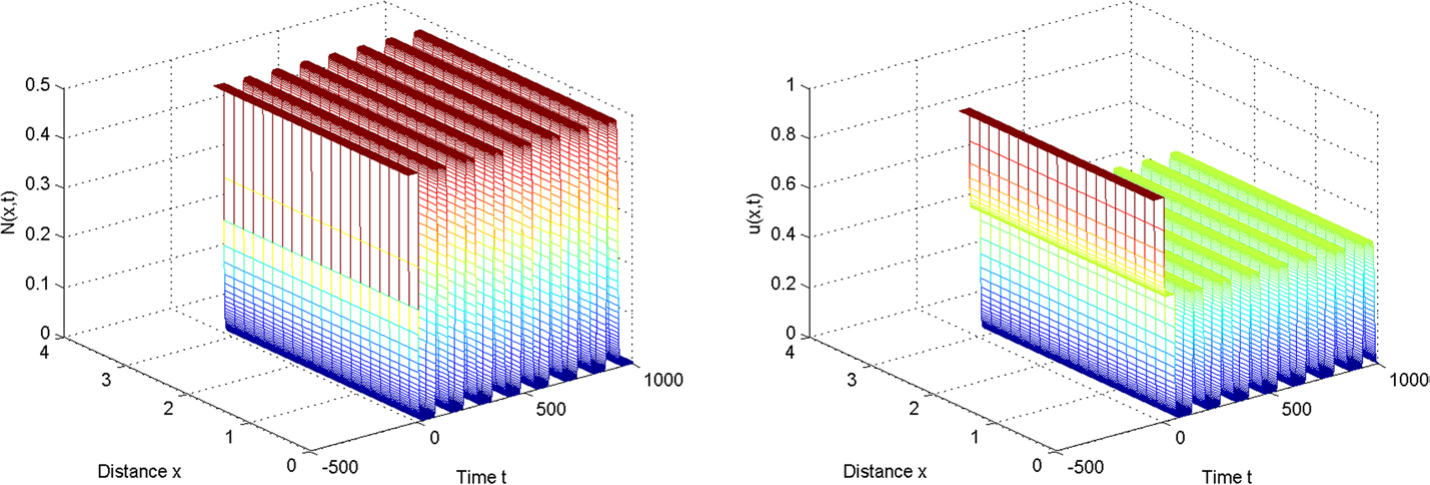
The spatially periodic solution still exists when $\tau =50$

**Fig. 4:**
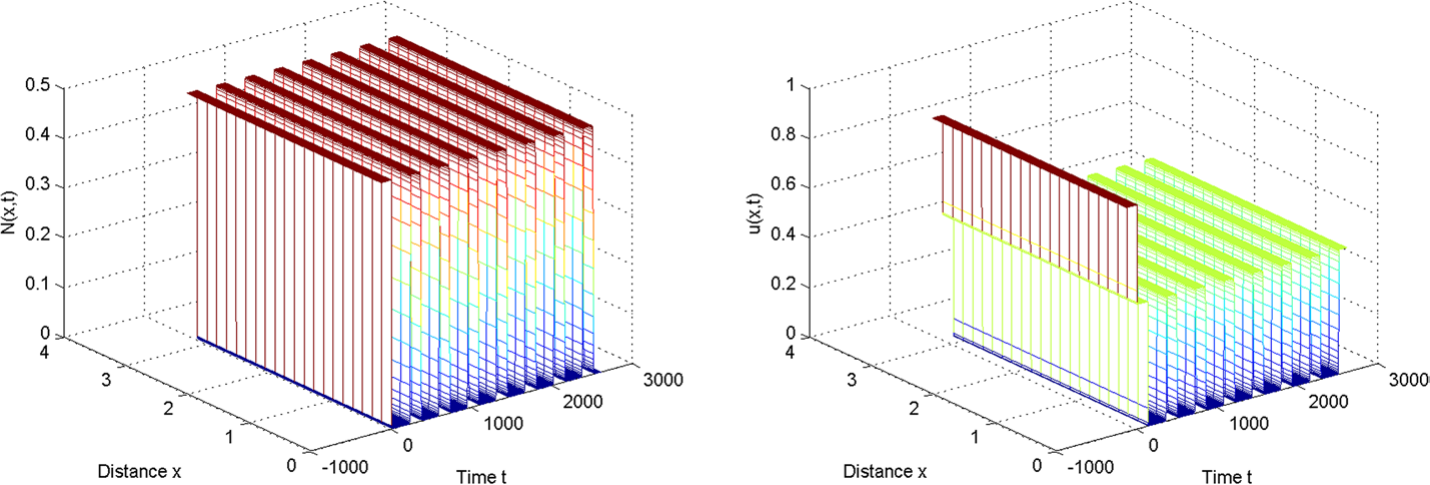
The spatially periodic solution still exists even when $\tau =130$

In the light of these simulations, we can find that spatially periodic solutions still exist even when $\tau =50\in (\tau _2^{(0)}, \tau _3^{(0)})$ and $\tau =130\in (\tau _6^{(0)},\tau _7^{(0)})$ (see Figs. 3, 4).

## Discussions and conclusions

In this paper, we considered the reaction–diffusion regulated logistic growth model. We have investigated the basic properties and Hopf bifurcation under the Neumann boundary conditions. It is shown that the logistic model may undergo Hopf bifurcation when time delay varies. We further give the formulae for determining the bifurcation properties, such as the direction of bifurcation, the stability of periodic solution and the monotonicity of period of periodic solution.

Here, we only discussed the single–species diffusive model with feedback control. In fact, how spatial diffusion and time delay affect the dynamic behaviors of multi–species controlled model remains unclear. We will focus on these novel and interesting models in the future.

Furthermore, from the numerical simulations in section “Numerical simulations”, we conjecture that the Hopf bifurcation induced by time delay is global. This means that the periodic solutions due to Hopf bifurcation still exist even if the time delay is sufficiently large.
